# Supplementary material for: Vaccine Hesitancy: COVID-19 and Influenza Vaccine Willingness among Parents in Wuxi, China—A Cross-Sectional Study
Source: Vaccines (Basel). 2021 Apr 1;9(4):342. doi: 10.3390/vaccines9040342 (PMC8066309; doi:10.3390/vaccines9040342)
Supplement: Supplementary file 1 [file vaccines-09-00342-s001.zip › supplementary material1.pdf]

Content

Figure S1 Wuxi city in China
Figure S2 Areas and Counties in Wuxi city
Table S1 Vaccine hesitancy scale scores by participant’s socio-demographics (N= 3009)
Figure S3 Parental acceptance of vaccination against COVID-19 and influenza for child
Figure S4 Reasons being willing to accept vaccine against COVID-19
Figure S5 Reasons being not willing to accept vaccine against COVID-19
Table S2 Comparisons to COVID-19 and influenza vaccine acceptance by socio-demographics (N=3009)
Table S3 Relationship between vaccine hesitancy with COVID-19 and influenza vaccination acceptance
Figure S6 Distribution of responses to 10-item of the vaccine hesitancy scale for healthcare workers
Figure S7 Healthcare workers’ acceptance of vaccination against COVID-19
Figure S8 Reasons being willing to accept vaccine against COVID-19 for healthcare workers
Figure S9 Reasons being not willing to accept vaccine against COVID-19 for healthcare workers

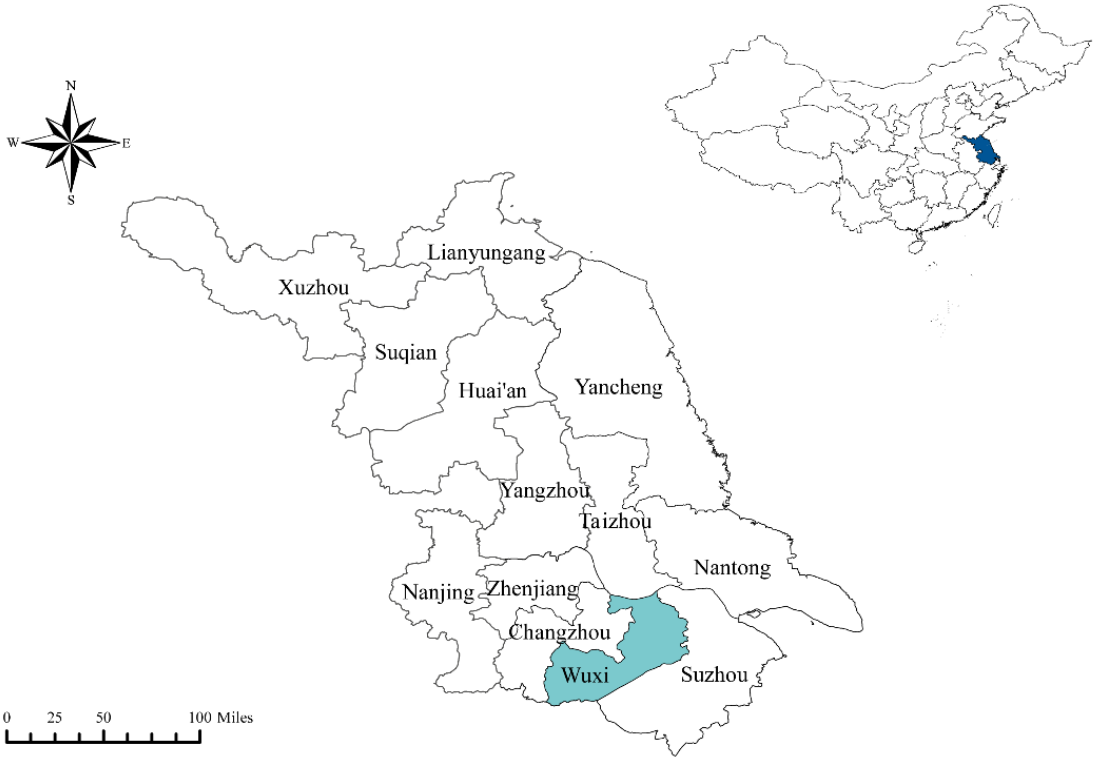

Figure S1 Wuxi city in China

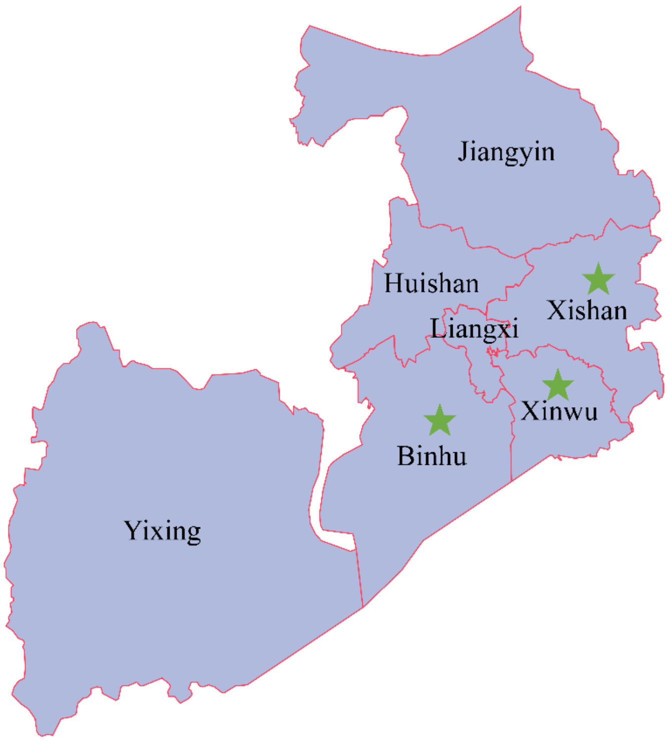

Figure S2 Areas and Counties in Wuxi city*

*Level A (>US$26 billion) included Jiangyin county and Xinwu area; level B (US$15–26 billion) included Yixing county, Liangxi area, and Binhu area; and level C (< US$15 billion) included Huishan area and Xishan area (http://yw.tj.wuxi.gov.cn:8081/pc/), the green star represented the selected area.

Table S1 Vaccine hesitancy scale scores by participant's socio-demographics (N= 3009)

Socio-demographics	Category A VHS scores	Category B VHS scores	P value
Total	40.96±3.87	38.58±5.05	0.000
**Children**			
**Age group**			
<1	41.17±3.82	38.66±5.13	0.000
1 –<2	41.07±4.15	38.83±5.03	0.000
2 –<3	41.17±3.98	38.18±5.42	0.000
3 –<4	40.31±3.87	38.26±4.67	0.000
4 –<5	40.64±3.60	38.53±4.68	0.000
5 –<6	41.00±3.47	38.56±5.09	0.000
≥6	40.82±3.83	38.70±5.10	0.000
P value	0.021	0.330	
**Sex**			
Male	41.0±3.94	38.64±4.99	0.000
Female	40.8±3.79	38.52±5.11	0.000
P value	0.696	0.678	
**Firstborn**			
No	41.20±3.97	38.48±5.24	0.000
Yes	40.83±3.80	38.64±4.95	0.000
P value	0.021	0.182	
**Parents**			
**Relationship with child**			
Mother	40.85±3.73	38.45±5.00	0.000
Father	41.28±4.24	38.96±5.18	0.000
P value	0.009	0.011	
**Age group**			
< 26	40.65±3.97	38.19±5.41	0.000
26 –<31	41.05±3.78	38.68±4.98	0.000
31 –<36	40.92±3.77	38.56±5.01	0.000
36 –<41	40.90±4.29	38.50±5.15	0.000
≥41	41.38±3.70	39.09±4.93	0.000
P value	0.599	0.559	
**Educational level**			
Junior high school or below	40.84±4.05	38.19±5.40	0.000
High school graduate or equivalent	40.74±3.98	38.36±5.08	0.000
College or equivalent	41.01±3.77	38.68±4.95	0.000
Master's Diploma or above	41.26±4.06	38.89±5.23	0.000
P value	0.183	0.054	
**Annual household income (10,000 RMB)**			
<5	40.53±4.24	37.99±5.28	0.000
5 –<10	41.20±4.02	38.71±5.05	0.000
10 –<15	40.91±3.77	38.42±4.93	0.000
≥15	40.88±3.73	38.70±5.08	0.000
P value	0.152	0.170	
**Healthcare occupation**			
Yes	41.47±3.62	39.00±4.84	0.000
No	40.92±3.88	38.55±5.06	0.000
P value	0.178	0.324	

VHS: vaccine hesitancy scale

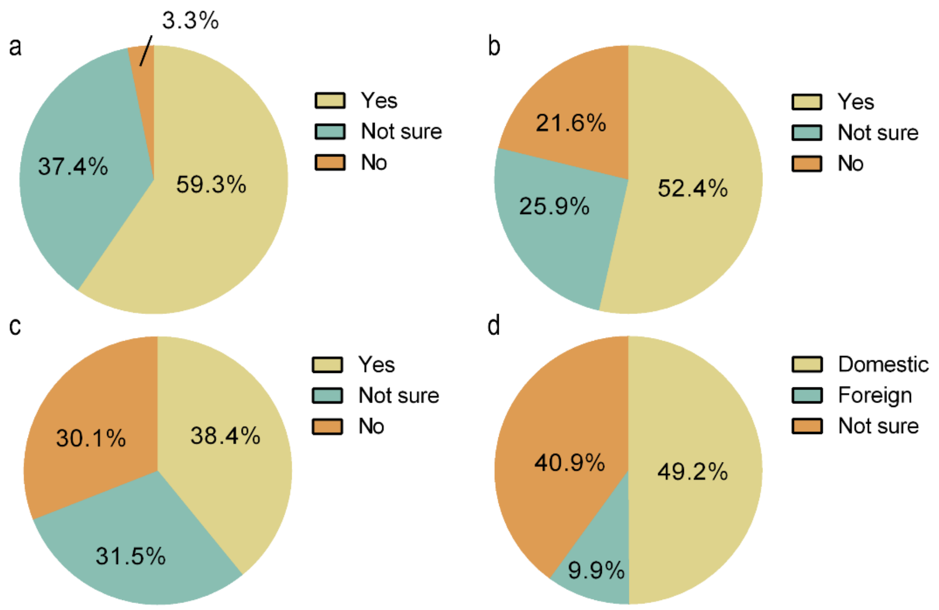

Figure S3 Parental acceptance of vaccination against COVID-19 and influenza for child†

†a: Question: If COVID-19 vaccine is available, will you vaccinate for your child? b: Question: will you vaccinate your child against influenza this year? c: Question: Will you be vaccinated against COVID-19 for your child as the first population? d: Question: Will you choose domestic or foreign COVID-19 vaccine for your child?

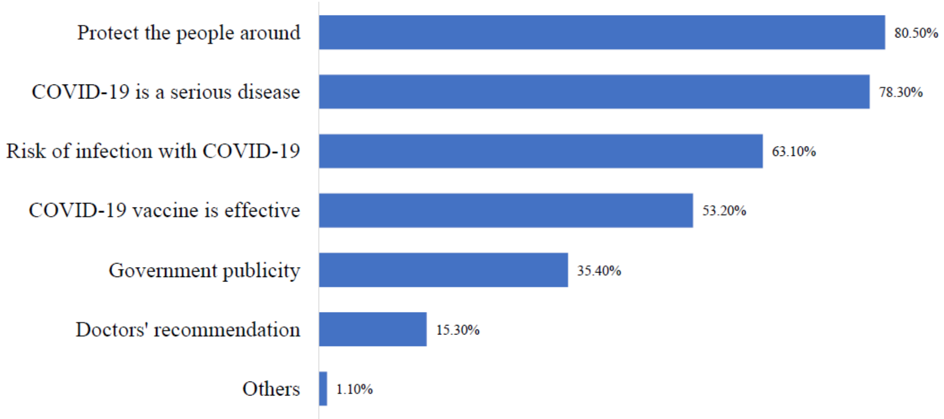

Figure S4 Reasons being willing to accept vaccine against COVID-19 for parents

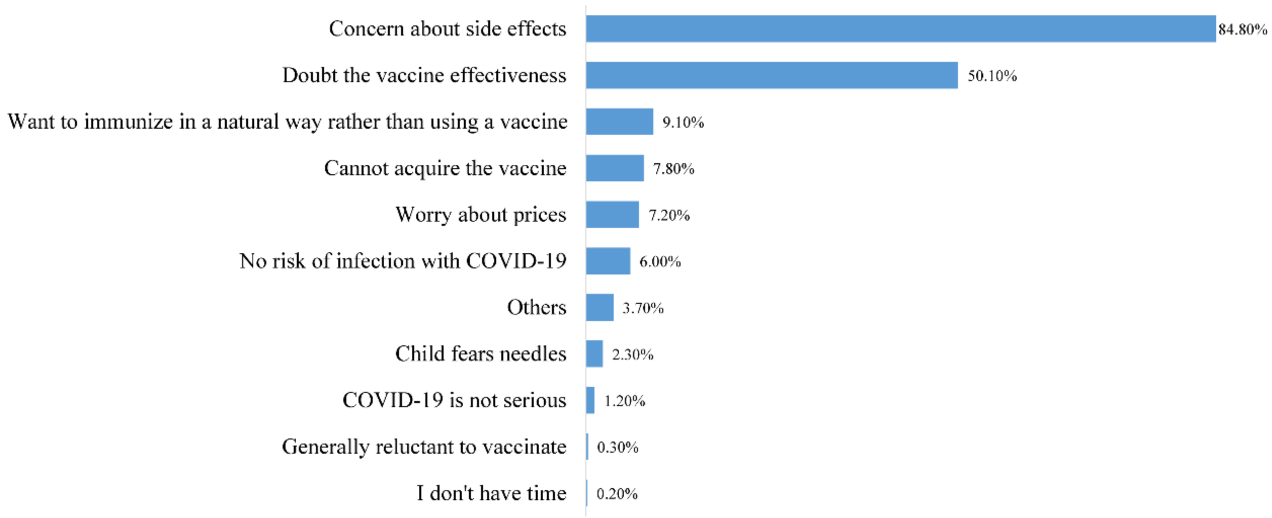

Figure S5 Reasons being not willing to accept vaccine against COVID-19 for parents

Table S2 Comparisons to COVID-19 and influenza vaccine acceptance by socio-demographics (N=3009)

Socio-demographics	COVID-19 vaccine acceptance		Influenza vaccine acceptance	
	Yes (N=1793)	Not Sure and No (N=1216)	Yes (N=1611)	Not Sure and No (N=1398)
**Children**				
**Age group**				
<1	599(56.1%)	468(43.9%)	484(45.4%)	583(54.6%)
1 –<2	287(61.5%)	180(38.5%)	230(49.3%)	237(50.7%)
2 –<3	198(61.7%)	123(38.3%)	136(42.4%)	185(57.6%)
3 –<4	183(61.4%)	115(38.6%)	206(69.1%)	92(30.9%)
4 –<5	186(59.4%)	127(40.6%)	196(62.6%)	117(37.4%)
5 –<6	93(65.5%)	49(34.5%)	102(71.8%)	40(28.2%)
≥6	247(61.6%)	154(38.4%)	257(64.1%)	144(35.9%)
P value	0.139		0.000	
**Sex**				
Male	909(59.5%)	619(40.5%)	793(51.9%)	735(48.1%)
Female	884(59.7%)	597(40.3%)	818(55.2%)	663(44.8%)
P value	0.911		0.067	
**Firstborn**				
No	610(59.4%)	417(40.6%)	527(51.3%)	500(48.7%)
Yes	1183(59.7%)	799(40.3%)	1084(54.7%)	898(45.3%)
P value	0.877		0.078	
**Parents**				
**Relationship with child**				
Mother	1341(59.7%)	904(40.3%)	1235(55.0%)	1010(45.0%)
Father	452(59.2%)	312(40.8%)	376(49.2%)	388(50.8%)
P value	0.781		0.006	
**Age group**				
< 26	152(61.0%)	97(39.0%)	100(40.2%)	149(59.8%)
26 –<31	678(62.1%)	413(37.9%)	541(49.6%)	550(50.4%)
31 –<36	682(58.7%)	480(41.3%)	655(56.4%)	507(43.6%)
36 –<41	226(54.2%)	191(45.8%)	257(61.6%)	160(38.4%)
≥41	55(61.1%)	35(38.9%)	58(64.4%)	32(35.6%)
P value	0.069		0.000	
**Educational level**				
Junior high school or below	209(59.9%)	140(40.1%)	156(44.7%)	193(55.3%)
High school graduate or equivalent	378(67.1%)	185(32.9%)	283(50.3%)	280(49.7%)
College or equivalent	1099(58.5%)	779(41.5%)	1045(55.6%)	833(44.4%)
Master's Diploma or above	107(48.9%)	112(51.1%)	127(58.0%)	92(42.0%)
P value	0.000		0.000	
**Annual household income (10,000 RMB)**				
<5	122(57.3%)	91(42.7%)	102(47.9%)	111(52.1%)
5 –<10	555(63.4%)	321(36.6%)	434(49.5%)	442(50.5%)
10 –<15	475(60.9%)	305(39.1%)	394(50.5%)	386(49.5%)
≥15	641(56.2%)	499(43.8%)	681(59.7%)	459(40.3%)
P value	0.009		0.000	
**Healthcare occupation**				
Yes	114(61.3%)	72(38.7%)	95(51.1%)	91(48.9%)
No	1679(59.5%)	1144(40.5%)	1516(53.7%)	721(25.5%)
P value	0.625		0.487	
**Influenza vaccination acceptance**				
Yes	1109(68.8%)	502(31.2%)	-	

No	684(48.9%)	714(51.1%)	-
P value	0.000		-

Table S3 Relationship between vaccine hesitancy with COVID-19 and influenza vaccination acceptance

Vaccine hesitancy scale		COVID-19 vaccine acceptance					Influenza vaccine acceptance				
					Correlation					Correlation	
		Yes (N=1793)	Not Sure and No (N=1216)	P value	value	P value	Yes (N=1611)	Not Sure and No (N=1398)	P value	value	P value
Category A	Total scores*	40.96±3.89	40.95±3.84	0.689	-0.007	0.689	40.95±3.85	40.97±3.89	0.947	-0.001	0.947
	L1	4.87±0.40	4.82±0.44	0.000	0.064	0.000	4.86±0.40	4.83±0.43	0.024	0.041	0.024
	L2	4.82±0.45	4.76±0.49	0.000	0.069	0.000	4.82±0.44	4.77±0.49	0.002	0.056	0.002
	L3	4.82±0.48	4.76±0.53	0.000	0.070	0.000	4.82±0.49	4.77±0.51	0.001	0.061	0.001
	L4	4.75±0.57	4.63±0.66	0.000	0.097	0.000	4.74±0.57	4.66±0.65	0.000	0.075	0.000
	L5	2.92±1.31	3.04±1.17	0.004	-0.052	0.004	2.92±1.30	3.02±1.20	0.021	-0.042	0.021
	L6	4.61±0.66	4.47±0.72	0.000	0.103	0.000	4.60±0.67	4.5±0.704	0.021	0.080	0.000
	L7	4.74±0.55	4.63±0.59	0.000	0.112	0.000	4.72±0.56	4.66±0.57	0.000	0.068	0.000
	L8	4.69±0.66	4.62±0.64	0.000	0.079	0.000	4.68±0.64	4.63±0.66	0.007	0.049	0.007
	L9	2.61±1.32	2.80±1.23	0.000	-0.081	0.000	2.63±1.29	2.75±1.28	0.006	-0.050	0.006
	L10	2.14±1.28	2.42±1.27	0.000	-0.123	0.000	2.15±1.26	2.38±1.30	0.000	-0.096	0.000
Category B	Total scores*	38.92±4.87	38.08±5.26	0.000	0.074	0.000	39.34±4.57	37.71±5.42	0.000	0.157	0.000
	L1	4.44±0.82	4.20±0.93	0.000	0.136	0.000	4.52±0.76	4.15±0.95	0.000	0.209	0.000
	L2	4.47±0.77	4.26±0.85	0.000	0.128	0.000	4.53±0.73	4.22±0.87	0.000	0.193	0.000
	L3	4.49±0.78	4.27±0.88	0.000	0.137	0.000	4.56±0.72	4.22±0.91	0.000	0.203	0.000
	L4	4.27±0.89	3.99±0.96	0.000	0.147	0.000	4.32±0.87	3.97±0.97	0.000	0.187	0.000
	L5	2.80±1.16	2.98±1.04	0.000	-0.089	0.000	2.81±1.19	2.95±1.01	0.001	-0.068	0.000
	L6	4.40±0.79	4.17±0.86	0.000	0.138	0.000	4.44±0.76	4.16±0.88	0.000	0.166	0.000
	L7	4.45±0.79	4.24±0.86	0.000	0.133	0.000	4.51±0.74	4.20±0.88	0.000	0.188	0.000
	L8	4.33±0.91	4.08±0.97	0.000	0.141	0.000	4.39±0.86	4.05±1.00	0.000	0.182	0.000
	L9	2.75±1.21	2.99±1.10	0.000	-0.102	0.000	2.78±1.21	2.93±1.13	0.001	-0.068	0.000
	L10	2.50±1.28	2.89±1.19	0.000	-0.162	0.000	2.49±1.27	2.85±1.22	0.000	-0.149	0.000

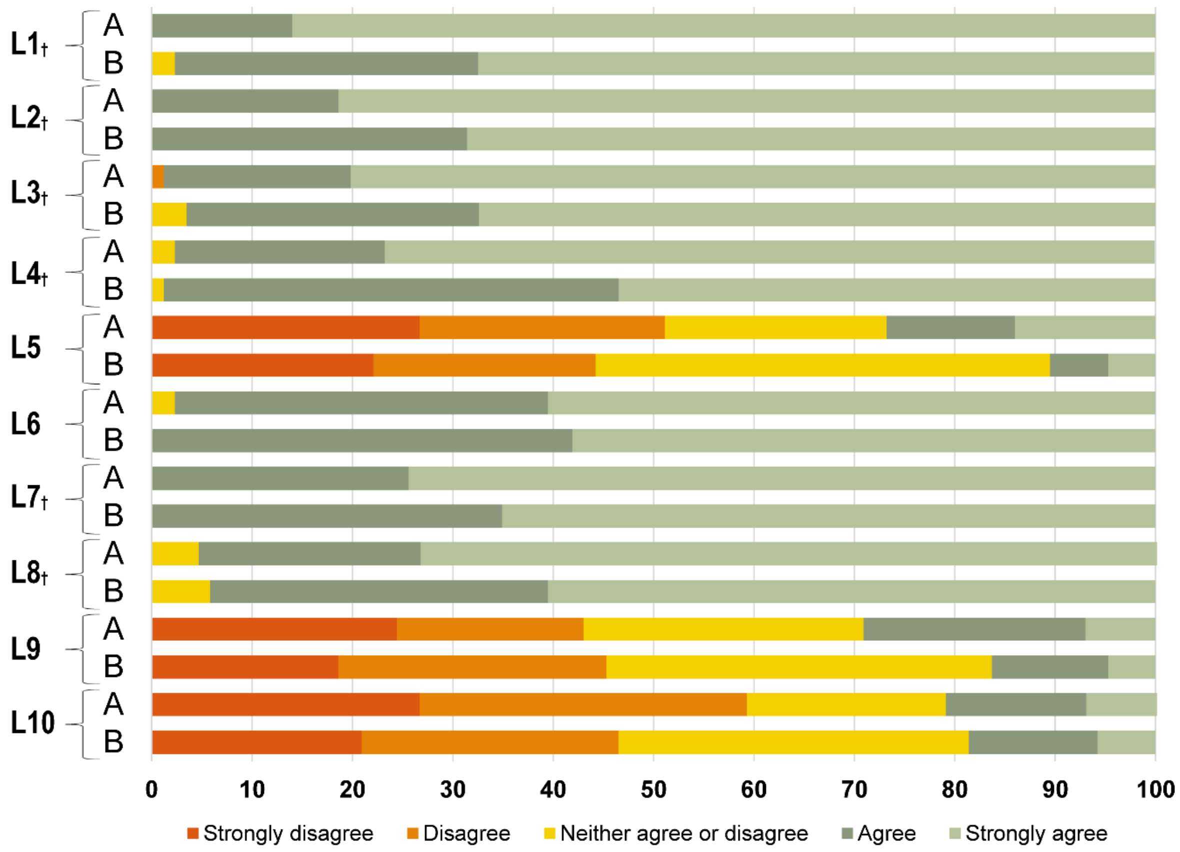

Figure S6 Distribution of responses to 10-item of the vaccine hesitancy scale for healthcare workers

† $P<0.05$, A represent category A vaccine, B represent category B vaccine, L1: Childhood vaccines are important for my child's health, L2:Childhood vaccines are effective, L3:Having my child vaccinated is important for the health of others in my community, L4:All childhood vaccines offered by the government programme in my community are beneficial, L5:New vaccines carry more risks than older vaccines, L6:The information I receive about vaccines from the vaccine program is reliable and trustworthy, L7:Getting vaccines is a good way to protect my child/children from disease, L8:Generally I do what my doctor or health care provider recommends about vaccines for my child/children, L9:I am concerned about serious adverse effects of vaccines, L10:My child/children does or do not need vaccines for diseases that are not common anymore.

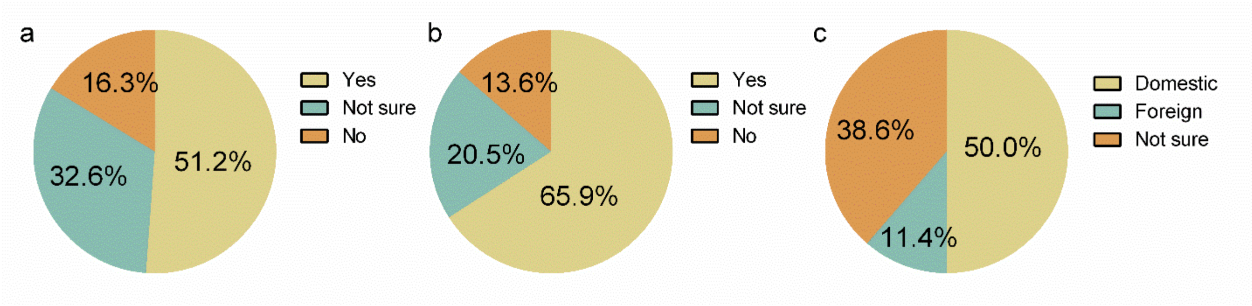

Figure S7 Healthcare workers' acceptance of vaccination against COVID-19†

†a: Question: If COVID-19 vaccine is available, will you vaccinate for yourself? b: Question: Will you be vaccinated against COVID-19 as the first population? c: Question: Will you choose domestic or foreign COVID-19 vaccine?

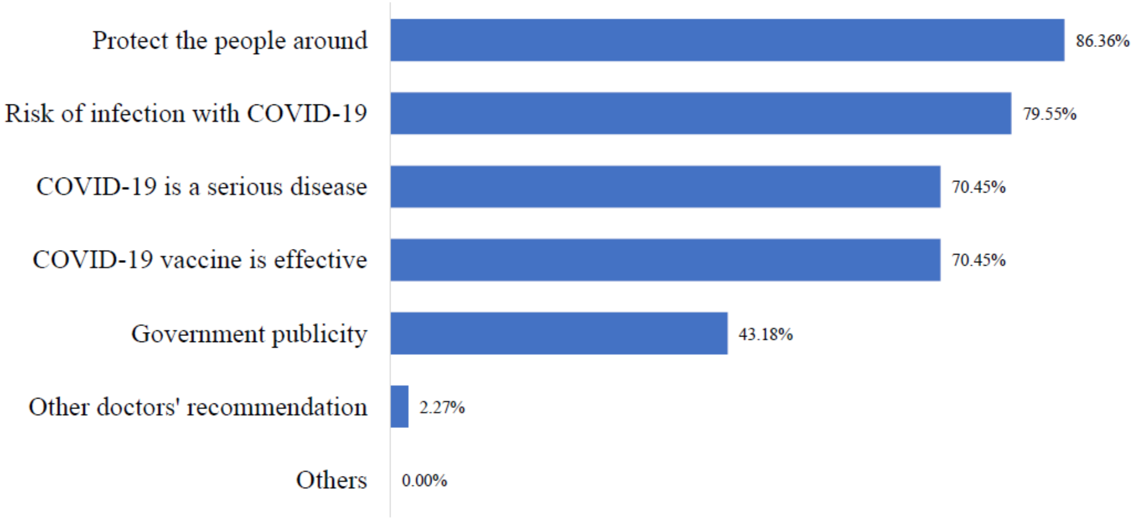

Figure S8 Reasons being willing to accept vaccine against COVID-19 for healthcare workers

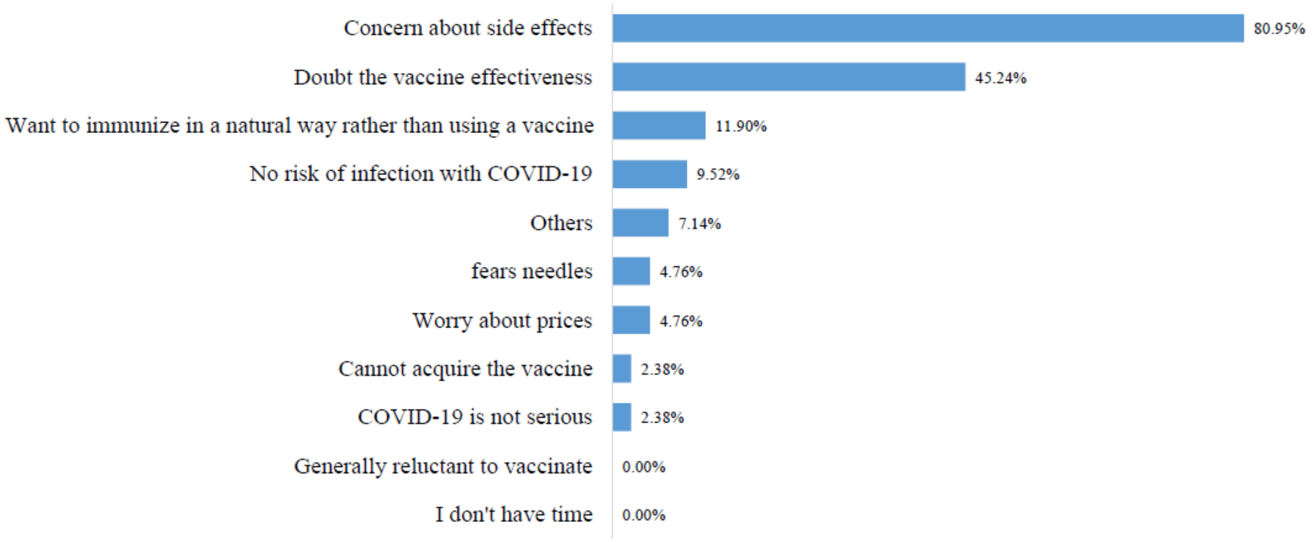

Figure S9 Reasons being not willing to accept vaccine against COVID-19 for healthcare workers
