# Supplementary material for: Vaccine Hesitancy: COVID-19 and Influenza Vaccine Willingness among Parents in Wuxi, China—A Cross-Sectional Study
Source: Vaccines (Basel). 2021 Apr 1;9(4):342. doi: 10.3390/vaccines9040342 (PMC8066309; doi:10.3390/vaccines9040342)
Supplement: Supplementary file 1 [file vaccines-09-00342-s001.zip › supplementary material2.pdf]

## Method

We performed an exploratory factor analysis (EFA) and confirmatory factor analysis (CFA) to assess reliability and validity of vaccine hesitancy scale (VHS). All analyses were conducted in SPSS 23.0 and AMOS 23.0. We randomly split the sample into two parts, one for EFA and another for CFA.

We reported the KMO value and Bartlett's test of sphericity to examine correlations between 10 items. Principle component analysis was performed to group those items together into factors that will maximize the variance in the data using a measure known as an eigenvalue. We retained only the factors that have eigenvalues of more than 1.0, and then used Kaiser normalization varimax rotation to identify if any of the items might be redundant within a factor.

We used CFA to confirm that whether each latent factor was being measured properly by each item. The index Chi square/DF($\chi 2$/DF), root mean square error of approximation (RMSEA), goodness-of-fit index (GFI), adjusted goodness-of-fit index (AGFI), comparative fit index (CFI), Tucker-Lewis index (TLI), and standardized root mean square residual (SRMR) were used to evaluate the model fit. We calculated average variance extracted (AVE) and composite reliability (CR) to assess the degree of internal consistency among the items of each latent factor, namely convergent validity. The square root of AVE and correlations between latent factors to assess the discriminant validity.

In addition, we calculated Cronbach's $\alpha$ to determine internal consistency.

## Results

The sample size was 1504 in EFA, and 1505 in CFA. The characteristics in two samples were not significantly different (Table S1). In category A VHS, the Bartlett's test of sphericity ($p < 0\ .001$) showed that correlations between items were sufficient to conduct an EFA. Two factors were identified with eigenvalues more than one through EFA. Two factors explained 62.41% of the common variance of the 10 items scale (Table S2). Factor 1

explained more common variance than factor 2. There are seven items loading on Factor 1, and they are primarily related to vaccine confidence; there are three items loading on factor 2, related to vaccine risk and complacency. Similar results have shown in category B VHS.

In category A VHS, model fit was acceptable according to a series index (Table S3). For factor 1, factor loadings to four items were greater than 0.7 and three items were lower than 0.7 but greater than 0.6. The AVE of 0.5463 (>0.5) and CR of 0.8928 (>0.7) which indicated that the convergent validity was good (Table S4). However, the convergent validity for factor 2 need to been improved. The correlation between two latent factors was significant and was lower than the square root of AVE, which indicated the discriminant validity was good in category A VHS (Table S5).

In category B VHS, model fit need to been improved (Table S3). For factor 1, factor loadings to six items were greater than 0.7 and one items were lower than 0.7 but greater than 0.6. The AVE of 0.6579 (>0.5) and CR of 0.9303 (>0.7), which indicated that the convergent validity was good. Similarly, the convergent validity for factor 2 need to been improved. The correlation between two latent factors was significant and was lower than the square root of AVE, which indicated the discriminant validity was good in category B VHS.

In category A VHS, the factor 1 consisted of seven items with Cronbach's $\alpha$ of 0.875, and the factor 2 consisted of three items with Cronbach's $\alpha$ of 0.681. In category B VHS, the factor 1 consisted of seven items with Cronbach's $\alpha$ of 0.907, and the factor 2 consisted of three items with Cronbach's $\alpha$ of 0.706.

**Table S1 Responders' characteristics in EFA sample and CFA sample (N= 3009)**

| Characteristic | | EFA Sample (N= 1504) | CFA Sample (N= 1505) | P value |
|---|---|---|---|---|
| **Children** | | | | |
| **Age, y (Mean ± SD)** | | 2.23 ± 2.43 | 2.25 ± 2.50 | 0.990 |
| **Age group** | | | | |
| | <1 | 533 | 534 | 0.966 |
| | 1 to <2 | 235 | 232 | |
| | 2 to <3 | 160 | 161 | |
| | 3 to <4 | 153 | 145 | |
| | 4 to <5 | 148 | 165 | |
| | 5 to <6 | 70 | 72 | |
| | ≥6 | 205 | 196 | |
| **Gender** | | | | |
| | Male | 757 | 771 | 0.623 |
| | Female | 747 | 734 | |
| **Firstborn** | | | | |
| | Yes | 509 | 518 | 0.739 |
| | No | 995 | 987 | |
| **Parents** | | | | |
| **Relationship with child** | | | | |
| | Mother | 1126 | 1119 | 0.746 |
| | Father | 378 | 386 | |
| **Age, y (Mean ± SD)** | | 31.24 ± 4.44 | 31.49 ± 4.47 | 0.309 |
| **Age group** | | | | |
| | < 26 | 135 | 114 | 0.173 |
| | 26 to <31 | 550 | 541 | |
| | 31 to <36 | 576 | 586 | |

| | | | |
|---|---|---|---|
| 36 to <41 | 208 | 209 | |
| ≥41 | 35 | 55 | |
| **Educational level** | | | |
| Junior high school or below | 186 | 163 | 0.288 |
| High school graduate or equivalent | 264 | 299 | |
| College or equivalent | 943 | 935 | |
| Master's Diploma or above | 111 | 108 | |
| **Annual household income (10,000 RMB)** | | | |
| <5 | 110 | 103 | 0.220 |
| 5 to < 10 | 428 | 448 | |
| 10 to <15 | 413 | 367 | |
| ≥15 | 553 | 587 | |
| **Healthcare background** | | | |
| Yes | 1414 | 1409 | 0.653 |
| No | 90 | 96 | |

**Table S2 Results of EFA analysis**

| Item | Category A | | Category B | |
|---|---|---|---|---|
| | Factor 1 loading | Factor 2 loading | Factor 1 loading | Factor 2 loading |
| L1 | 0.825 | 0.045 | 0.841 | 0.103 |
| L2 | 0.839 | 0.065 | 0.867 | 0.069 |
| L3 | 0.824 | 0.079 | 0.873 | 0.054 |
| L4 | 0.787 | 0.079 | 0.761 | 0.089 |
| L5 | -0.007 | 0.763 | -0.020 | 0.808 |
| L6 | 0.707 | 0.108 | 0.724 | 0.101 |
| L7 | 0.813 | 0.064 | 0.846 | 0.095 |
| L8 | 0.707 | -0.001 | 0.702 | 0.104 |
| L9 | 0.134 | 0.797 | 0.126 | 0.827 |

| | | | | |
|---|---|---|---|---|
| L10 | 0.065 | 0.787 | 0.164 | 0.738 |
| **Eigenvalue** | 4.368 | 1.872 | 4.574 | 1.938 |
| **Variation** | 43.683 | 18.725 | 45.739 | 19.381 |
| **KMO** | 0.881 | | 0.890 | |
| **Bartlett's test of sphericity** | 0.000 | | 0.000 | |

**Table S3 CFA model fit**

| Scale | X2/df | RMSEA | GFI | AGFI | IFI | TLI | SRMR |
|---|---|---|---|---|---|---|---|
| Category A VHS | 3.879 | 0.044 | 0.967 | 0.946 | 0.877 | 0.834 | 0.0624 |
| Category B VHS | 6.450 | 0.060 | 0.945 | 0.942 | 0.853 | 0.804 | 0.0682 |
| Suggested value for good fit | 2–5 | <0.06 | >0.90 | >0.90 | >0.90 | >0.90 | <0.08 |

**Table S4 Convergent validity of different VHS ***

| Scale | Item | Factor | Estimate loading | AVE | CR |
|---|---|---|---|---|---|
| Category A | L1 | Factor 1 | 0.762 | | |
| | L2 | Factor 1 | 0.791 | | |
| | L3 | Factor 1 | 0.816 | | |
| | L4 | Factor 1 | 0.646 | 0.5463 | 0.8928 |
| | L6 | Factor 1 | 0.659 | | |
| | L7 | Factor 1 | 0.840 | | |
| | L8 | Factor 1 | 0.628 | | |
| | L10 | Factor 2 | 0.618 | | |
| | L9 | Factor 2 | 0.723 | 0.4208 | 0.6839 |
| | L5 | Factor 2 | 0.598 | | |
| Category B | L1 | Factor 1 | 0.867 | 0.6579 | 0.9303 |
| | L2 | Factor 1 | 0.918 | | |

| | | | | |
|---|---|---|---|---|
| L3 | Factor 1 | 0.836 | | |
| L4 | Factor 1 | 0.743 | | |
| L6 | Factor 1 | 0.751 | | |
| L7 | Factor 1 | 0.842 | | |
| L8 | Factor 1 | 0.698 | | |
| L10 | Factor 2 | 0.682 | | |
| L9 | Factor 2 | 0.760 | 0.4977 | 0.7476 |
| L5 | Factor 2 | 0.671 | | |

*The following cutoff criteria were used: (a) AVE >0.5, (b) CR > 0.7

**Table S5 Discriminant validity of different VHS**

| Scale | | Factor1 | Factor2 |
|---|---|---|---|
| Category A | Factor1 | 0.739 | |
| | Factor2 | 0.183* | 0.827 |
| Category B | Factor1 | 0.811 | |
| | Factor2 | 0.270* | 0.705 |

*P<0.001

**Table S6 Internal consistency of different VHS**

| Value | Total sample | | | |
|---|---|---|---|---|
| | Category A | | Category B | |
| | Factor 1 | Factor 2 | Factor 1 | Factor 2 |
| Cronbach's $\alpha$ | 0.875 | 0.681 | 0.907 | 0.706 |
